# Feminization, rural transformation, and wheat systems in post-soviet Uzbekistan

**DOI:** 10.1016/j.jrurstud.2022.03.025

**Published:** 2022-05

**Authors:** Dina Najjar, Rachana Devkota, Shelley Feldman

**Affiliations:** a Social, Economics and Policy Research Group, International Center for Agricultural Research in the Dry Areas (ICARDA), Avenue Mohammed, Bearabi Alaoui, Agdal Hay Ryad, Instituts Maroc, Rabat, Morocco; b School of Environmental Design and Rural Development, University of Guelph, 50 Stone Road E, ON, N1G 2W1, Canada; c Feminist, Gender & Sexuality Studies Program, Cornell University, 319 East Court Street, Ithaca, NY, 14850, USA

**Keywords:** Gender, Feminization of agriculture, Wheat innovation, Post-soviet agriculture, Out-migration, Uzbekistan

## Abstract

This paper examines how rural transformation in Uzbekistan alters gender norms and roles and, consequently, affects women's involvement in agriculture. We focus on the role that contextual factors, particularly kinship relations, government goals, and institutional structures each contribute to rural transformation and male out-migration, and how these, in turn, increase women's work in wheat production and processing. The wheat is the most important crop in the country which has the highest area coverage (35%) in Uzbekistan. We begin by highlighting the post-Soviet transition in Uzbekistan and its effects on the agricultural sector, including how households respond to opportunities for innovation. We then move to a discussion of our methodological approach drawing on insights from the GENNOVATE project, a collaborative initiative across 11 CGIAR centres that explored the relationship between changing gender norms in relation to women's roles in agricultural production and processing. Next, we examine an understudied topic in migration research i.e., how the transformation of agriculture contributes to increased dependence on unpaid female agricultural labour. We conclude with an analysis of how the feminization of agriculture alters household relations and women's participation in the public sphere. Significantly, we close with a reflection on what these changes mean for gender and innovation studies.

## Highlights

• We examine how rural transformation in Uzbekistan alters gender norms and roles.
• We found that the transformation of agriculture increased women's unpaid labour.
• In migrant communities women gained confidence and made demands for equal treatment.
• Gender norms are still restrictive but that they are changing among mothers-in-law.
• Policy changes alone are insufficient to realize changes in gender expectations.

## 1 Introduction

This paper examines how rural transformation in post-Soviet Uzbekistan contributes to shaping women's involvement in agricultural production and processing. We focus on the role that contextual factors, particularly kinship relations, government goals, and institutional structures each contribute to rural transformation and how these, in turn, recast women's involvement in wheat production and processing, the single most important crop in the country occupying the largest area of production (FAO 2020). Much has been written about wheat production in Uzbekistan from a macroeconomic and technological perspective (e.g., Morgounov et al., 2001; IFPRI, 2010; Kienzler et al., 2011; Djanibekov et al., 2012; Rudenko et al., 2012; Sharma et al., 2013). However, exploring the gendered implications of wheat production on local livelihoods and well-being has garnered far less attention (see Kandiyoti, 2003; Gunchinmaa et al., 2011; Tursunova, 2012; 2013 as exceptions). Also, in the agriculture and innovation sector, gender dynamics and relations have received far too little attention (Badstue et al., 2017). This is the case despite the fact that as we know, capacity building and innovation have been popular foci of development agencies, funding bodies, and development policy since the turn of the 21 Century (The World Bank, 2007; IFPRI 2010), and have contributed to the documentation of the benefits, means, and opportunities for innovation and improvement in the agricultural sector which are acknowledged as highly gendered. However, despite these achievements, there continues to be a lack of attention to the intersection of gender norms and resulting impacts on the adoption of, and adaption to, agricultural innovations (Badstue et al., 2017; Gunchinmaa et al., 2011).

For example, there has been limited attention to changes in gender roles and norms more broadly in response to rural transformation in Uzbekistan, or, more specifically, to the impacts of these changes on agriculture. In this paper, we follow this transition by examining how this transformation impacts gender norms in wheat systems and, consequently, how women fare in their ability to farm, adopt, and improve upon agricultural innovations in wheat production. We build on the recognition of scholars from other world regions that there is far too little understanding of the impacts of rural transformation on gender norms and supports, as well, the need to understand such impacts as context-dependent (De Haas and Van Rooij, 2010; Ressurecion & Van Khanh, 2007; De Haas & Fokema 2010; Abdelali-Martini and Dey de Prick, 2015). The role of contextual factors, such as government policy and the changing role of mothers-in law is also considered in four case studies from four districts of Uzbekistan: Jondor in Bukhara, Kamashi in Kashkadarya, Pazdargom in Samarkand and Boz in Andijan. In these four study sites we address three research questions: 1) How has rural transformation impacted gender norms in agricultural systems in the districts? 2) What are the key contextual factors that have contributed to rural transformation outcomes related to the feminization of agricultural production? and 3) How have gender norms reshaped agricultural adaptations and innovations in wheat production?

We begin by highlighting the post-Soviet transition in Uzbekistan and its effect on the agricultural sector with a focus on wheat production. We then move to a discussion of our methodological approach and draw on insights from the GENNOVATE project, a collaborative initiative across 11 Consultative Group on International Agricultural Research (CGIAR) centres that explored the relationship between changing gender norms in relation to women's roles in agricultural production and processing. We follow this with a discussion of the understudied topic of rural transformation. This includes attention to the shift to self-sufficiency in wheat production policies, land reforms, and male-outmigration which together have contributed to an increased dependence on unpaid family, usually female, agricultural labour. Finally, we provide an analysis of how this transformation alters household relations and women's participation in the public sphere. We argue that migrant households are important cites of changes in gender relations and show how these contextual changes increased the workload of household members and led to precarious working conditions. Significantly, we close with a reflection on what these changes mean for gender relations, rural transformation, and innovation among households.

## 2 Transition economy and feminization in agriculture in post-soviet Uzbekistan

### 2.1 An overview of the transition economy in rural areas

Uzbekistan, located in Central Asia, was formally part of the Soviet Republic which became independent in 1991. With the collapse of the USSR, the Uzbek state was forced to consider ways to increase income sources as the country lost Soviet funding (Kandiyoti, 2003; Djanibekov et al., 2012). Public sector employees lost their jobs due to the state's lack of funding and the state turned to agriculture to provide a readily available means to employ the population (Kandiyoti, 2003; Djanibekov et al., 2012). Independent Uzbekistan is largely dependent on agriculture which accounts for 25 percent of the GDP and employs 32 percent of the population. Cotton production is the country's most significant source of revenue, accounting for 50 percent of export earnings with up to 99 percent of earnings in some provinces (Kandiyoti, 2003; Rudenko et al., 2012).

With the breakdown of the soviet state, markets were disrupted and Uzbekistan faced a grain and flour deficit (Kandiyoti, 2003; Djanibekov et al., 2012). The state aimed to reduce wheat imports, sustain profits from cotton production, and employ its population through individually run farms and expanding the area of existing plots (Kandiyoti, 2003). During this post-independence period, a Decree of the Cabinet of Ministers stipulated achieving self-sufficiency in wheat production (Rudenko et al., 2012). Under this system, wheat production increased from 940,000 tonnes to 8 million tonnes in 2015 with yield also increasing from 26051 kg/ha in 2000–47821 kg/ha in 2014 (World Grain, 2015).

To accomplish these goals, Shirkats or collective farms were organized into family-based farms and allocated specific acreage on a contract basis. The area was allocated based on family size and crops cultivated that, for the most part, focused on wheat and cotton, the two crops for which the state provided credit at three per cent interest and dictated a quota for collection at the end of the season (Kandiyoti, 2003). The state also enabled access to subsidiary plots, the ability to secure inputs for household and subsidiary plots, including fuel wood from cotton stalks, and access to grazing land for livestock (Kandiyoti, 2003). A few Shirkats were also distributed to private or independent farmers who still had to meet state quotas but who had some independent decision-making power in land and input use and the marketing of their crops (Djanibekov et al., 2012). Eventually, the Shirkats devolved into private farms with a preferred size of 15 ha until all the land was distributed (Djanibekov et al., 2012). Table 1 details the stages of farm restructuring in post-Soviet Uzbekistan.

**Table 1:** Farm restructuring stages in post-Soviet Uzbekistan.

Stages/years	Major reform	Transformation process	Dominant farm types	Policy objectives
First stage 1992–1997	Decollectivization of state farms	Transformation of *sovkhozes (state farms)* into *kolkhozes (collective farms)*	*Kolkhozes, Sovkhozes*	Expansion of wheat area & yields, reorganization of state farms
Second stage 1998–2002	Farms Partial fragmentation	Transformation of *kolkhozes* into *shirkats*. Land lease to individual farms	*Shirkats*, individual farms	Specialization of newly established individual farms
Third stage 2003–2008	Complete fragmentation	Complete transformation of *shirkats* into individual farms	*Shirkats*, individual farms	Development of non-cotton/wheat producing sectors, and livestock farms
Fourth stage 2008/09-2015	Farm consolidation	Farm reconsolidation (farm-size optimization)	Individual farms, mainly cotton-grain producers	Increased and stable cotton yields, relocation of cotton fields
Fifth stage 2016-present	Production specialization	Fragmentation and optimization of production	Individual farms of different specialization	Relocation of cotton and wheat fields, increased area of high value crops, multi-profile farms

More recently, in 2008, under an ‘optimization’ program, private farmlands were drastically reduced and consolidated into larger areas averaging 33–90 ha, a pattern similar to pre-independence times (Djanibekov et al., 2012; Jumaev, 2011). The rationale for this consolidation was to increase efficiency and synchronize farming scale with the infrastructure inherited from the Soviet period (Djanibekov et al., 2012). The procurement system for wheat and cotton persisted throughout this reform and since 2005 (Rudenko et al., 2012). Many independent farmers lease their lands to multiple families to whom they pay a monthly salary, often to the head of the household (Tursonova et al., 2012; Kandiyoti, 2003). As Tursunova (2012) reports, there are two different arrangements among independent farmers, one that entails a monthly payment and/or an additional small plot which is the more common arrangement, and a second that entails sharecropping on a 50-50 basis.

Kandiyoti (1998, 2003) argues that the agrarian reform introduced a set of contradictory measures for agriculturalists. On the one hand, the Uzbek State was under pressure from international donors to privatize land and, on the other hand, the State was keen to control the production of wheat and cotton. While cotton is a revenue generating crop, wheat is a staple crop which incurs a hefty importation bill. Consequently, different land tenure systems were introduced: privately owned small plots or *tomorka,* commercial farms tied to the State through leaseholds, and contracts with farmers who previously were either independent or members of *Shirkats*.

Dekhans (smallholders or peasant farmers) received a subsidiary plot of about 0.13ha, and although each household was entitled to private land, in provinces where population density was high, this was not possible (Kandiyoti, 2003). Of our four sites, Andijan is the most densely populated followed by Samarkand (Bektemirov and Rahimov, 2001) where families used these plots to grow subsistence crops and produce goods for barter as a way to mitigate the costs of unemployment, late payment, meagre wages, and diminished social protection by the state. An FAO report (2019) indicated that in labour law, women who work in dekhan farms do not receive any kind of protection (such as sick pay, maternity leave or childcare leave). The small-holder economy and the state-led export economy were dependent on one another (Kandiyoti, 1996; Kandiyoti, 2002, 2003) even as land distributed to Dekhans is inheritable and bequeathable and was not affected by subsequent land reform measures (Djanibekov et al., 2012).

**Fig. 1:**
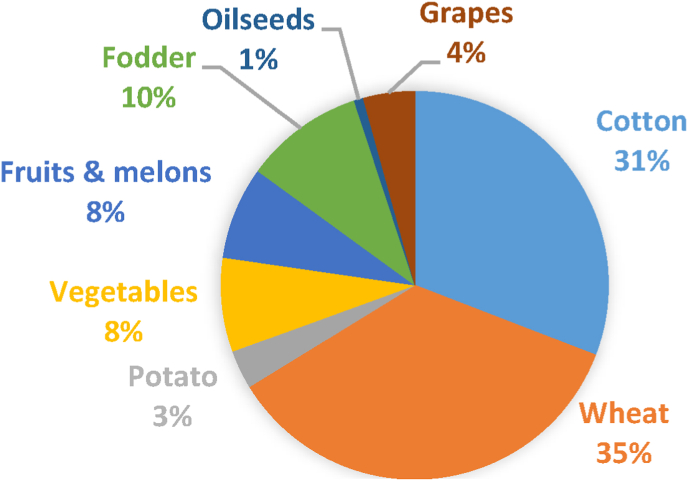
Projected sown area (’000 ha) of crops in Uzbekistan in 2020. Data Source: World Bank Group, 2019

**Fig. 2:**
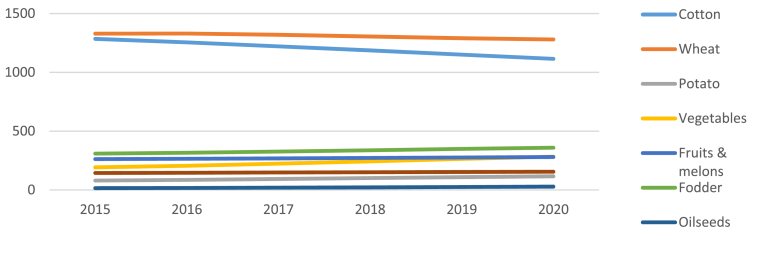
Projected sown area (’000 ha) of crops grown in Uzbekistan from 2015 to 2020. Data source: World Bank Group, 2019

While wheat and cotton are controlled by the state, vegetable, feed, and other crops are sources of subsistence and income (Kandiyoti, 2003; Djanibekov et al., 2012). However, the area cultivated with wheat has continued to increase at the expense of fodder crops (Djanibekov et al., 2012). Furthermore, water and land are largely allocated to wheat and cotton crops leaving limited resources for the cultivation of cash crops or rearing livestock (Djanibekov et al., 2012; Rudenko et al., 2012). Fig. 1, Fig. 2 reveal that wheat and cotton are the dominant crops in the country with cotton witnessing a decrease in the past five years. Vegetables and fruits occupy far less area and are witnessing a slight increase in area planted during the past five areas.

### 2.2 Feminization in agriculture in post-soviet Uzbekistan

The feminization of agriculture is characterized as the increased involvement of women in agriculture, either as family workers, independent producers, or wage workers in non-traditional agro-export production (Lahiri-Dutt, 2014; Pattnaik et al., 2017). It also refers to women's ownership of farmland and resources (Agarwal 2012), labour (Tamang et al., 2014), decision making power (Lastarria-Cornhiel, 2008), and the recognition of their contributions in the public domain which were previously considered men's sphere (Mandel 2013; Deere 2005). Research addressing the concept of the “feminization of agriculture” has been carried out in both developed and developing countries (Lahiri-Dutt, 2014; Deere 2005; Pattnaik et al., 2017; Agarwal 2012; Lastarria-Cornhiel, 2008) and often associated with different factors of rural transformation, such as changes in political and rural economic processes, male labour outmigration, the increasing number of female-headed households, and the rise of labour-intensive agriculture (Mukhamedova and Wegerich, 2018; Kelkar, 2009). In the context of Uzbekistan, during and after the Soviet period, women's roles in agriculture as family workers, entrepreneurs, or wage labourers have similarly changed, raising a set of issues to which we now turn.

The Uzbekistan Socialist Republic during the Soviet period was an agrarian republic employing 40 percent of the labour force in the agricultural sector (Kandiyoti, 2002). In this period, agricultural production was focused on crop-specialized, large-scale collective and state-owned farms (sovkhoz and kolkhoz). Furthermore, the Soviet system endeavoured to manage gender relations by employing men and women in production on a similar basis, resulting in the relative equal participation of men and women on collective farms (Mukhamedova and Wegerich, 2018) and motivating woman to participate in economic and political decision-making processes (Mickiewicz, 1977). However, traditional family structure coupled with Islamic customs generally considered women caretakers and supporters of men while men were assumed to be breadwinners and heads of the family (Kandiyoti, 2002). Some researchers (Akiner, 1997; Kandiyoti, 2002) argued that gender roles have not changed within the family, even in post-Soviet Uzbekistan. But, with the collectivization of private farms, women have been assigned wage employment, thereby redefining the traditional role of women as housewives and dependents of men (Constantine, 2007).

However, some governance aspects of the Soviet period related to agriculture continued to exist, including the *Mahallas* (where people apply for land) and Women Committees. Some argued that the *Mahallas* continued to be a means for the state to implement its policies and programs, expanding their role to include distributing aid to needy families (Bektemirov and Rahimov, 2001; Kandiyoti, 2003) while others view them as a post-independence means to devolve decision-making power to communities (see Bektemirov and Rahimov, 2001) and offer salaries to Kandiyoti executive members of *Mahalla* committees (Bektemirov and Rahimov, 2001). A governance innovation post-soviet times included the introduction of Water User Associations (WUA) to devolve decision-making related to water distribution and maintenance of infrastructure to local communities (Gunchinmaa et al., 2011).

These changes dramatically transformed rural life with some arguing that the gender dynamics of the transition led to a regression in patriarchal attitudes, including an increased dependence on women's informal labour and their confinement to the household (Kandiyoti, 1998, 2003; Tursunova, 2012; Lastarria-Cornhiel and Garcia-Frias, 2005; Gunchinmaa et al., 2011). In her exploration of the outcomes of different types of farm restructuring and work, Kandiyoti (2003), for example, finds that mechanization limited work opportunities especially for women. The transition to family-based farms also increased women's participation in agriculture but without recompense (Kandiyoti 1998, 2003). While women labourers were occasionally hired for low wages or payment in kind, or were engaged in reciprocal labour exchanges, these changes mark a dramatic contrast with the Soviet period where they received fixed salaries and social benefits (Kaniyoti 2003; Gunchinmaa et al., 2011).

Research on independent women farmers in Andijan, Navbakhor, and Khorezm provinces reveal that individually leased land was usually distributed to former administrative officials (Kandiyoti, 2003; Gunchimnaa et al., 2011; Tursunova, 2012). As few women held such positions, they were unable to access large holdings as independent farmers and those who did gain such access were wives of men in administrative and technical positions whose networks proved invaluable in securing land. Husbands of these women, however, often managed their land because it was largely perceived to be men's responsibility.Studies conducted in Andijan and Khorezm also reveal that women farmers on private farms have difficulty managing their farms given the need to meet quotas set by the state, delayed payments, and large landholdings (Kandiyoti, 2003; Gunchimnaa et al., 2011; Tursunova, 2012). In Khorezm, for instance, women's work burden increased when they became private farmers as they have to meet multiple responsibilities including leadership as well as farming and household labour demands. Thus, women responded by negotiating terms and conditions for farming their leased land with working families, labourers, and the state (Tursunova, 2012). Women's burden is further complicated as they face increased male-outmigration even as they enjoy increased, if limited, participation and decision-making power on water distribution committees (Gunchimnaa et al., 2011).

Although agriculture was able to absorb the unemployed population, population growth and meagre wages and opportunities fuelled both the internal and international out-migration of women but, more significantly, of men (FAO 2019; Malyuchenko, 2015, Tursunova, 2012; Lastarria-Cornhiel and Garcia-Frias, 2005; Rios, 2006). According to the Asian Development Bank (ADB) (2014), Uzbekistan is the topmost sending country in central Asia as migrants leave to the Russian Federation and Kazakhstan where they share language and family connections and benefit from central Asia's liberal visa requirements (IOM, 2006). Seven percent of the economically active population in Uzbekistan is involved in internal or external migration, 60–90 percent of whom are male (Al, 2014) even as female migration in response to limited work opportunities has also increased (FAO 2019; Bondarenko 2021). These work opportunities were in the domain of agriculture for women and construction for men. Findings from Khorezm province, for example, reveal that the sale of horticultural products in Russia and Kazakhstan was a thriving niche for women, while men worked in construction (Tursunova, 2011).

During the Soviet period, women were also employed in education and health but, as the state lost its funding, those able to keep their jobs received either in-kind payment or low wages. Others were put on unpaid leave although they continued to benefit from social protection measures such as paid maternity leave while engaging in informal trade and services (Kandiyoti, 2003; Lastarria-Cornhiel and Garcia-Frias, 2005). Not much has changed since the transition, and rural women's unemployment rates remain high (Turnsonova, 2012). While there have been limited studies on the impact of post-Soviet policies and subsequent changes in women's agricultural roles in the face of more restrictive gender norms (e.g., Kandiyoti, 2003; Tursunova, 2012; Mukhamedova and Wegerich, 2018), even fewer studies address the effects of male out migration on gender norms and women's roles in agriculture (Gunchinmaa et al., 2011), a topic to which we now turn.

We define norms as the formal and informal rules and codes of conduct that define appropriate behaviour and structure human interactions (North, 1991; Bruton et al., 2010). It is crucial, however, to recognize that normative prescriptions are not homogenous, and that normative behaviour is not doctrinal (Feldman, 2010). Rather, normative expectations mark relations of gendered expectations that vary across and within ethnic and religious groups as well as by country, class, and economic status. Also important to emphasize is that prescriptive behaviour not only defines that which is acceptable and that which is not, but norms often legitimate sanctions and thus serve to control social action and choice (Feldman, 2010). Importantly, too, when women and men do not abide by the rules of the game out of necessity or circumstances, they negotiate the space in which new norms emerge (Cornwall and Edwards, 2010). Poor women, for example, are often required to engage in labour relations that do not conform to normative expectations and while they may be sanctioned, their behaviour is often the site of negotiation where new normative consensuses emerge.

Examining changes in gender norms in Khorezm and Andijan provinces, our findings reveal that although Women's Committees' roles have been broadened in post-Soviet times, and now include providing aid to needy families, they focus exclusively on the domestic sphere, whereas earlier the state encouraged and sanctioned women's economic participation (Kandiyoti, 2003). Women's roles thus shifted to the informal sector and unpaid home gardens and subsistence production that reinforced more conservative male and female roles (Gunchinmaa et al., 2011; Al, 2014; Lastarria-Cornhiel and Garcia-Frias, 2005). Kandiyoti (2003) thus, unsurprisingly, argues that the focus of the state on the domestic sphere contributed to this conservative normative shift in women's roles as a generation of young Uzbekistan women are increasingly expected to comply with notions of domesticity (Kandiyoti, 2003).

Migration literature that looks at changes in gender roles and/or gender norms differentiates between permanent and temporary migration. In a study of female rural migration in Northern Vietnam, for example, although roles between men and women changed temporarily as women worked in the city and men undertook reproductive roles at home; with frequent return visits, women were able to reassure men that these roles were only temporary and that balance would be restored and gender norms would be reproduced following their migration (Resurreccion and Van Khanh 2007). Return migration from the Arab Gulf countries similarly restricted gender norms in Jordan (Tuccio and Wahba, 2015) where return migrants spread conservative gender norms related to women's mobility and participation in work they had adopted while abroad.

There also have been a few studies focused on the gender implications of migration as a consequence of agrarian reform (Hasanov and Sanaev, 2018) where, as Kandiyoti (1998) notes, male outmigration increased as a result of the transition economy in Uzebekistan. Tursunova (2013) explores outmigration of rural women and its impacts on women's health and livelihood in … but argues for the limited evidence available on how women who stayed behind sustained their participation in agriculture where their employment increased from 26 percent to 32 percent (Gunchinmaa et al., 2011). In Navbakhor province, despite women managing farms, their participation remains disproportional in Water User Associations (WUAs) since land is not registered in their names (Gunchinmaa et al., 2011). Gunchinmaa and his colleagues conducted their research in 2005–2007, prior to the initiation of optimization in 2008 that led to land consolidation and a reduction in the total number of farmersin agriculture (Djanibekov et al., 2012; Jumaev, 2011). In this paper, we address the post-optimization period, and while prior migration studies tend to focus exclusively on wives, we examine the impact of migration on mothers-in-law who play significant roles in the extended families that characterize the majority of our sample households. We focus on their complex relationships with other household members and whether and how, as women left behind, they were either empowered through wheat innovations, ignored, or not powerful enough to adopt innovations.

## 3 Methods

**Fig. 3:**
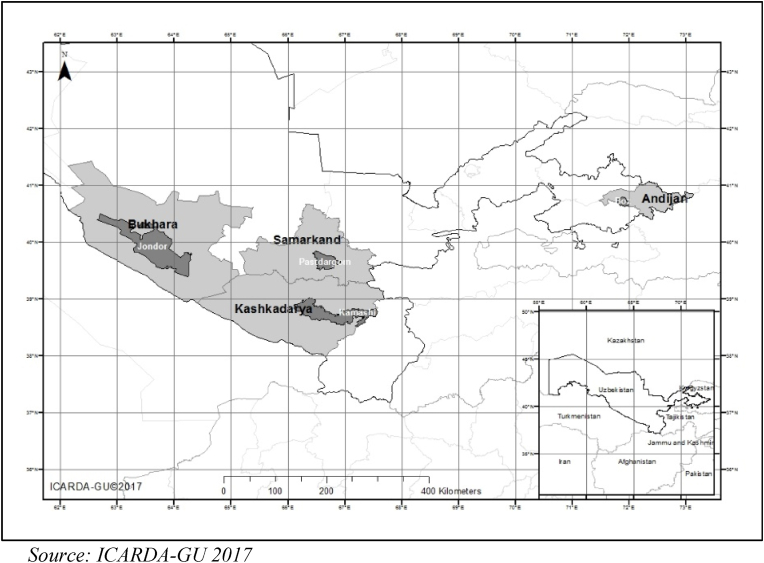
Provinces and districts of the four study sites. Source: ICARDA-GU 2017.

The data for this study is part of GENNOVATE, a global research project whose aim was to understand linkages between gender norms, agency, and capacities to innovate where interactions among gender, age, and social class were an explicit focus (Petesch et al., 2018). In 2014, 2015, and 2016 a series of case studies were conducted in four communities in Uzbekistan as part of the GENNOVATE project (Fig. 3). The four provinces, which exemplify the four sample communities, were selected based on prior survey results from a sample of 1400 households that addressed gendered decision-making power in the management of household farms (Yigezu 2012). This survey, conducted in 2012 in eight provinces, maximized diversity in decision-making power dynamics. Table 2 provides details from this survey for our four study communities (see Table 3).

**Table 2:** Decision-making power on household plots in four provinces.

Region	Sample size	Women manage the plot^a^ (in %)	Men manage the plot^a^ (in%)	Both equally manage the plot^a^ (in %)
Kashkadarya	280	22.1	77.9	0.0
Andijan	275	0.7	97.5	1.8
Bukhara	159	0.0	60.4	39.6
Samarkand	271	70.7	29.3	0.0

a The plot was defined here as the one closest to the house.

**Table 3:** Data collection details adopted for this study.

Data collection methods	Study sites (total no. of activities)				Sample size per activity	Total Sample size
	Kashkadarya	Andijan	Bukhara	Samarkand		896
Focus group discussions (FGDs) with the middle and poor classes farmers	2	2	2	2	10 participants in each FGD	800
Interviews with local innovators	4	4	4	4	2 men and 2 women	16
Life histories with successful innovator/farmers	4	4	4	4	2 men and 2 women farmers	16
Key informant interviews with leader	4	4	4	4	2 men and 2 women leaders	16
Interviews with workers	12	12	12	12	6 women and 6 men	48

Kashkadarya, for example, ranked lowest in women's participation in the joint management of plots closest to the house, but, unlike the communities in Andijan and Bukhara, showed that women manage farms alone in more than 20 percent of the cases. Kashkadarya is a dry province with poor land quality and livestock as the mainstay of the rural economy which may help to explain why agriculture was left for women to manage (Kandiyoti, 2003). In contrast, Andijan is a fertile province where agriculture is profitable and men manage almost all plots. Bukhara ranks highest in joint decision making, 39.6 percent, and Bukhara women are viewed as being the most liberal in the country as working outside the household is considered to be normal (Tursunova, 2013). In Samarkand, our last site, 25 percent of male temporary out migration suggests why there is high participation rates of women who manage plots closest to their house.

In each of the four communities we conducted two focus group discussions (FGDs) with eight to ten participants in each group. The groups included a women's and men's FGD from the middle and poor classes, and additional two youth groups similarly separating girls and boys in order to enhance the likelihood that people freely discuss their concerns. The rich, medium, and poor classes of farmers have been classified based on local authorities definitions of household types and resource ownership and access dynamics. Youth selection criteria include participants between 16 and 24 years old. All FGDs included at least six out of the eight to ten participants who were actively engaged in agriculture and represented diverse marital statuses and families. Separating groups by gender was assumed to enhance the likelihood that people could more freely discuss their concerns as was the decision to conduct interviews with local innovators.

In addition to the FGD, interviews were conducted with two men and two women local innovators in each community. Local innovators are defined as those farmers “who have developed their own agricultural varieties or management practices, or with local processors or vendors who have innovated in the local agricultural value chain” (Petesch et al., 2018, p. 98). Furthermore, life histories were also carried out with men and women farmers who were viewed as having improved their economic standing. These interviews shed light on understanding what people define as the conditions that led to either their success or failure to adopt wheat innovations. Sensitive issues, including household ownership and control over resources, were discussed in one-on-one interviews. Key informant interviews were also held with a man and woman leader in each community to provide a window on general community characteristics including women's participation in public life, average size of land holding, and the development programs that were available for women and men in their community. Lastly, in addition to this GENNOVATE data, twelve interviews were held with workers in each of the four communities, six women and six men, where discussion focused on the specific paid roles and the problems workers faced in agriculture, as well as their sources of agricultural, off-farm, and non-farm income.

The selection of study communities was not only based on prior relationships with researchers in the region but also where agricultural innovations had been introduced. In our sample communities -- Jondor in Bukhara, Kamashi in Kashkadarya, Pazdargom in Samarkand and Boz in Andijan – we had prior local contacts with the Ministry of Agriculture and Water Resources (MoAWR). The community in Kashkadarya also was selected because of ongoing activities in conservation agriculture at the time of fieldwork. These prior relations offered the GENNOVATE project baseline data as well as contributed to easing access to respondents who met the criteria of this study. After our interviews, responses were transcribed and translated into English, and coded in NVivo 10. Coded data were analysed using NVivo by categorizing the evidence into different themes.

## 4 Results and discussion

### 4.1 Rural transformation and feminization in agriculture in the four communities

In our study, farmers mentioned the re-consolidation of land in the post-Soviet period as a way to achieve efficiency gains for those able to fulfil state quotas. However, such land consolidation often led both male and female smallholder farmers to lose their land when used as collateral for loans they were unable to repay. This was a common theme among poor farmers in our study areas which suggests that the introduction of neoliberal reform is double-edged, it provides loans while also creating conditions that lead to land consolidation and loss among primarily smallholder farmers. Similarly, our findings showed that state credits are provided only for cotton and wheat production and machinery purchase that led to a focus on large producers. One farmer shared that “for other purposes, we can hardly get credit or loans from banks as they require pledges, and we have nothing valuable to pledge” (Naiman-C-M). This exclusive focus on wheat and cotton limited profits for farmers, and access to water and other resources available for other crops and livestock.

These conditions has led to a decline in the agriculture sector's ability to absorb the unemployed. For example, agricultural employment declined from 41.9 percent in 1991 to 33.4 percent in 2003, and then to 21 percent in 2009 (Tursunova, 2012; Sutton et al., 2013). FGD discussions with poor men in Samarkand illustrates this point, “ten years ago men had jobs only in agriculture, now many of them migrate to cities for construction jobs”. For those men currently involved in agriculture there was a sense that state rules do not encourage their production. As one Naiman farmer commented, “I tried to be a farmer and requested to get land, but it was rejected twice by khokimiyat” (UZ-Naiman-G2-M). Agriculture is not very attractive to young men; as one male youth from Naiman shared, “now, very few young men are interested in agriculture, as land is limited and state plans are tough to fulfil, too much controlled by the state” (UZ-Naiman-E-M). Unsurprisingly, many men in rural communities have resorted to migration in search of employment opportunities since, after independence, the collapse of the collective farm limited employment opportunities for both men and women. As Falkingham and Klytchnikova (2006) confirm, the economic transition in post-Soviet countries was a gendered phenomenon as the absence of men increased women's workload including providing opportunities for women and girls to participate in the labour market. As one interviewee commented: “I think that outmigration of male population of our *makhalla* forced us to be active. During last ten years, most men from our community migrated to Russia because they could not find the good job here” (UZ-Shodmonov-D-F).

This is a common voice among women in our four study sites as both permanent and temporary migration is very common in all study communities, where men migrate to large centres within Uzbekistan or to Russia for employment. Male temporary outmigration ranges from between 25 percent of households in Bukhara and Samarkand to 50 percent in Andijan and 75 percent in Kashkadarya with permanent male outmigration ranging between almost none in Bukhara and Samarkand to 25 percent in Andijan and 50 percent in Kashkadarya.^1^ Key informants as well as interview data confirm this finding. Focus groups discussions with male and female youth confirm that women are stay in rural areas as few migrate with their husbands and migration on their own is frowned upon as it implies illicit behaviour.

Women in all four communities reported having to work longer hours for a pay and in informal occupations given their meagre wages and the lack of opportunities for their husbands. Even among those whose husbands had migrated, they explained that the remittance income they receive is inconsistent and insufficient. Crucially, women's income generation opportunities differ by class, education, and other contextual factors shaping their daily lives, a point we detail in the next section. But, for now, when women were asked in Andijan about what leads households to fall into poverty, there was a consensus that migration has led to family breakdown and a significant cause of poverty. Men confirm this understanding and it was also evident in discussions among youth in Samarkand who emphasized the declining wages in Russia making migration less lucrative than it was in the past. One male farmer from *Naiman* also mentioned that “in Russia it was easier to make money before, but now many young men return as the exchange rate is not good as it used to be even in summer” (UZ-Naiman-E-M). Nonetheless, migration continues to be described as the most popular route for accumulating enough funds for marriage among residents in all four study communities. As one male farmer notes: “young men are eager to migrate as it is the only way to make enough money fast to be able to pay for a wedding and build one's own house” (UZ-Yortepa-E-M). Conversations with local residents in Andijan, for example, where migration, especially among the poor, is second highest among our study communities, estimated that the share of female-headed households is above the officially reported figure of five percent. Similar findings are reported in Tajikistan where male migration to Russia led to the abandonment of wives who must assume the role of provider for their family (Al Jazeera, 2014).

Complicating these gender differences in response to poverty is the role of mothers-in-law whose work burden increased with the migration of their sons and where, interestingly, they became more supportive of their daughter's in-law. There was general agreement in the four study communities that support from husbands and in-laws, and, in particular, mothers-in-law, was important in enabling women to take on farm management roles and succeed (see also Badstue et al., 2017). A woman in Andijan whose husband was initially sending money from Russia, then suddenly stopped and eventually abandoned his family, experienced significant changes in her relationship with her mother-in-law. The same mother-in-law was very critical of her daughter in-law when her son was around. The daughter in-law reported that criticism for her spending habits, including purchases of basic household items, “was the smallest insult.” She shares this to contrast her current situation when her mother-in-law transferred the title of the house to her because “she was ashamed of what her son had done …. In 2013 she wrote the house in my name so that my husband does not kick us out with the children. I respect her.”

Other examples of the changes in relations between daughters- and mothers-in-law similarly show that mother's in-law are more supportive towards their daughter in-law when their sons misbehave. As one married woman from Shodmonov shared during our interview: “my mother in-law now fully supports and permits me to do the business of selling bread. She registered the house in my name to show her support because she was angry at her son that he married in Russia without her permission and her son's action was shameful for her. She felt guilty toward me and my children”.

Middle class women in a FGD in Bukhara explained that women living with their mothers-in-law feel they have less power and freedom than living in a nuclear family because they need to seek permission from both their husbands and mothers-in-law. The relationship between middle-class mothers-in-law and daughters-in-law was reported by women and men alike to be problematic. To further illustrate the tension between daughters and mothers in law, a woman in Mugrak explains that the worst experiences in her life are “humiliation and abasement from mother's in-law”, but that with male outmigration, this relationship tends to change as mother's in-law's wellbeing becomes tied to the success of their daughters' in law. They thus become more supportive of each other.

Findings further indicated that out-migration does not always have a positive consequence as males who are unable to regularly send money to their family create tensions for women. In such situations, dependence on in-laws and male kin, including sons living in the household, was viewed as important in the lives of women who experience male outmigration. As they report, migration puts additional pressure on in-laws (parents of the migrant) and is well captured by an example from one Andijan woman's life history whose husband migrated to Russia: “Fortunately, my parents-in-law have got pensions. It saved us from family collapse. My husband sent us money only two or three times.” Similar findings of the increased burden placed on in-laws of male migrants are reported by Tursunova (2013). A woman who was farming on a large holding leased by the state to produce cotton and wheat on a commercial basis lost her land because her sons preferred to migrate rather than farm. This example contrasts with a woman whose husband passed away, but whose sons continued to farm the land along with their mother, indicating the variety of changes in household relations that have emerged in response to the agrarian transition.

### 4.2 Women's changing roles and the role of wheat innovation

As noted earlier, the post-independence emphasis on wheat farming led to an increase in production from 940,000 tonnes to 8 million tonnes (World Grain, 2015) and occupies the largest area of production. According to IFPRI 2010, wheat yields have increased nine-fold between 1991 and 2006 and Rudenko and his colleagues (2012) also report a continuous increase in the area under wheat production. According to the FAO report, the area under wheat cultivation increased by 62 percent between 1980 and 2012, from 500,000 ha in 1980 to 1.3 million hectares in 2012 (Lerman et al., 2016).These findings were confirmed in our FGDs with the youth in the four communities.

Wheat is largely a commercial crop. Although women's role in farming is increasing, as frequently reported in the sample communities and by national statistics, their ability to cultivate as independent farmers on a commercial basis has been limited. This is due to entrenched discrimination in the *Mahalla* (where people apply for land) and in the community (more details in next section). As such, very few women are able to access land for commercial wheat production even though focus groups across class and generation revealed that women as independent producers have been increasing in the past decade. Interviews with leaders in the community as well as with individual women revealed that most gained access to land through inheritance from their deceased husbands or fathers when titles were transferred to them. Unfortunately, after the death of their husbands, some women were unable to sustain yields to meet quota demands and thus lost their farms.

As discussed above, women's ownership and management of land has been increasing in all our study areas. However, findings also confirm limits on women's ability to manage their farm on a commercial basis and their dependence on family networks. One Samarkand woman whose husband passed away reveals that her sons preferred to migrate rather than engage in agricultural production. She subsequently fell ill and eventually lost the land. Another woman in Bukhara province whose husband works in the city registered her father's land in her name after her brother failed to farm the land due to alcohol abuse. He eventually passed away, but she felt obliged to return the land to her family. Another widowed woman consulted her father's male farmer friends about input prices because she was being charged two or three times more for services than her male counterparts. Despite this inequality, she reports that she continues to farm her father's land even as it significantly increases her workload. She is a lawyer in a government office and responsible for taking care of her mother as well as household chores. Moreover, she always needs to attend to the land because, as she explains, workers steal from her when she is at her second job and her nephew is in Russia and has refused to return to help her.

Among our sample population, women were more likely to be hired to manage a portion of a farmers' land or to sharecrop land where the farmer provides all the inputs and then shares equally in the produce. A woman farmer who was responsible for a farmer's land that was used to produce wheat for seed production for sale to the government, explained that while the income from this was higher than for wheat, she preferred grain because she could use it for household consumption. Very few women were able to rent land to cultivate wheat and other crops, such as watermelon and, in some areas, they reported that the land they were able to rent was of inferior quality and had limited access to irrigation. Women's more limited access to irrigation than men was reported in Bukhara, Samarkand and Kashkadarya but not in Navbakhor province highlighting important differences in gender discrimination (Gunchinmaa et al., 2011). Moreover, women farmers were unable to participate in the executive committee of Water User Associations and thus could not make important decisions regarding water allocation. Some middle-class women explained why they took leadership positions in these Associations: “male migration of our *Mahalla* (neighbourhood here) forced us to be active [albeit in limited leadership positions].”

Also, in Bukhara, limited rain and irrigation water has depressed wheat yield leading male farmers to prefer rearing livestock. For women, improved extension services in fruit and vegetable production are especially important as their role in cultivating fruits and vegetables has expanded in response to new markets and their need to diversify household income by farming on rented land as well as household plots. Women constitute three-fourths of the sellers in local markets in Bukhara, Adnijan and Kashkadarya and one half in Samarkand where they sell vegetables and baked goods.

Both men and the few women who farm wheat, often on a commercial basis, reported being able to learn about new wheat innovations from the *Mahalla* community, with men reporting that they have additional information networks including agricultural expos, farmers' cooperatives, and access to agronomists. But men also complained that agronomists only provided advice related to cotton and wheat and mentioned that they also need to resolve pest and agronomic issues related to vegetable and fruit crops. Although women's participation in public life increased, as reported by women in all four communities, their roles were largely limited to accountant, office manager, lawyer, and a member of the Women Committees, including president, rather than to positions with substantive decision-making power such as president of the *Mahalla,* a position almost exclusively occupied by men notwithstanding the increasing numbers who migrate.

We should also note that men were more likely than women to access credit to purchase agricultural machinery limiting women's ability to expand their enterprises. Women who did not usually produce primarily cotton and wheat were excluded from credit access which was largely limited to these crops. Men, however, resented the state's exclusive focus on cotton and wheat since they preferred to produce more lucrative cash crops where pests and sub-optimal practices were contributing to losses in yield in vegetables and fruits. The state also controls the organizational and technical issues associated with supplying machinery, fertilizer, fuel, and lubricating materials. After meeting contract obligations with the state, final payments are made to the government to fulfil their credit obligations. Some wheat farmers told us that during financial crises they rely on while women do not have this option because so few are wheat or cotton farmers. Nonetheless, women report resorting to informal credit sources such as forming groups and a rotating fund, known as gap (see also Kandiyoti, 1998). Middle class women in Bukhara explained that in addition to enabling them to purchase “something good” with small amounts of money, the gap was also important as “a cushion for black days.” For some women, the *Mahalla* committee provides credit to enable them to open bakeries of their own and to hire other women as well.

Other income diversification routes were reported in all four communities, including wage work in agriculture, where women are hired for harvesting and weeding, tasks that are reported to “require more patience and diligence.” Men are usually employed in machine-intensive tasks such as tractor driving and irrigation because “only men could water plots.” Our interviews with workers in the four provinces revealed that, on average, women earned 30 percent less than men in the wage sector (see also Gunchinmaa et al., 2011 for similar findings in Navbakhor province). Late payments were also reported as a concern by both women and men workers with some proposing that farmers could provide them with land for cultivating vegetables as in-kind payment instead of wages. In a FGD with poor women in Kashkadarya we also learned that the mechanization of cotton seeding reduced their employment options, a finding that Kandiyoti (2003), also found in Khrorezm and Andjian provinces.

From the perspective of the wheat value chain, women were far more likely to be involved in processing wheat by-products. Many field workers also reported that because men were paid in wheat, their wives had a readily available resource to diversify income by selling baked goods as petty traders along the roadside or in the market. Women confirmed this and acknowledged that newly opened private mills and bakeries created many job opportunities for them. These new relations reveal the increased commodification of products and labor even as it promotes traditional roles for women. A woman in Andijan, whose husband migrated to Russia and abandoned her, resorted to selling baked goods in the market and on the street with the help of her son who can push the kart for her. While she complains about severe weather, she appreciates that the local *Mahalla* has recently improved the market infrastructure. She also noted that she has been experimenting with different wheat varieties since she finds that local varieties are of poor baking quality while imported ones are superior and can fetch a higher price. A new wheat variety, *Yakshart,* which she learned about by talking with other women, enables her to purchase it in bulk and grind it at the mill, significantly reducing her costs of production. Here, meeting with other women reveals the importance of public engagement in helping women to transform their opportunities and abilities to secure their livelihoods.

There are serious quality concerns with locally produced wheat in Uzbekistan due to the tendency of mixing local product with higher quality, imported flour (Rudenko et al., 2012). For example, at the individual level, women use a similar strategy and mix imported wheat with locally produced wheat to reduce the price of baked goods. And, not surprisingly, men and women farmers prefer different characteristics in wheat varieties. Men's concerns include increased yield, resistance to disease, and early maturity, making their preferred innovation new wheat varieties. Women, on the other hand, prefer wheat-related innovations that include new mills, bakeries, and high yielding varieties with characteristics related to consumption. This finding shows that there are gender differences in the choices women and men make regarding the trait preferences in wheat variety. However, in all four communities, seed availability was the major problem where some farmers reported that they recycle their seeds three or four times. This might be the one of the reasons that women complain that planting material for wheat has many weeds in them.

### 4.3 Changes and continuity in gender norms and implications

Norms related to gender roles in the four communities reveal that men have the decision-making responsibility for wheat and all commercial crops, including livestock, while joint decision-making characterizes the production of subsistence crops and dairy products which are women's responsibility.^2^ Women whose husbands live with them seldom have exclusive decision-making power in any domain, but households where husbands and sons migrate are sites of changing gender norms. While women in these households make key economic decisions, it often comes at the cost of deteriorating economic conditions as households can neither diversify their income sources nor expand production. As one Bukharan woman explained, her son's migration after his father's death resulted in losing her husband's land. In fact, in all four communities, women across class and generation wished to halt male migration by addressing the need to create local job opportunities. As an example, middle class women in Bukhara reported, “we dream that our children stay with us and do not go to cities and abroad for work …. We do not want to live without husbands, and we do not want men to migrate.”

Whether women had migrant husbands or not, given low wage rates they had to work. In co-habiting households, men often controlled their income as they held responsibility for selling livestock, wheat, and cotton.

In non-migrant households, women grow agricultural products for everyday family subsistence and, if women had extra produce, they could sell it or, more likely, engage in barter. As a woman leader in Bukhara explains, “families can't grow all products on their own; as such, a woman goes to the village market and can exchange milk for oil or sugar … a woman is the cashier in the family.” Similarly, in Kashkadarya, poor women report that “because mainly we are responsible for food preservation and for financial expenses for food, we try to decrease the expenses for the flour, vegetables, and meat. We want to get these products from our household.” In both Mugrak and Andijan, young women in our FGD reported that while women help their husbands on their farms, men rarely help their wives on the garden plots (*tomorka*). However, women reported the importance of gathering their husbands' support in carrying out these activities. As reported by middle class women in Andijan, “because an Uzbek woman needs a husband's permission and support in all activities, even in a small business, she must inform him and get his agreement”. As the household head he has the final say in everything.

Although male migration enables women to transcend gender norms by managing household finances, they were still limited in the ways they did so. Their income activities continued to conform to domestic norms related to baking, vegetable farming and sales, as well as sewing. Yet the number of women becoming independent farmers and renting or sharecropping land is reported in interviews and FGDs to have increased in all four communities where being an independent farmer in Andijan was described as the “pinnacle of success.” In Kashkadarya, increased women's independence enabled them to join “the elite of a local community,” while in Samarkand it was described as the “best dream for our people,” since this change meant having a better income, an opportunity to acquire more land, and lease machinery. In a Bukhara focus group, poor women explained that the most challenging issue for them in moving up the social ladder is becoming an independent woman farmer, a position that conflicts with their reproductive roles. They also mentioned that husbands would be ridiculed if their wives became independent farmers which is also suggested by findings from Navabkor Province where women, in order to conform with gender norms, often register land in the names of their husbands even though they were farming the land (Gunchinmaa et al., 2011).

Despite growing in number, women managing farms as independent producers remains low. Yet, middle class women in Bukhara note that even when they are not registered as farmers, they are heavily involved in the management of family farms. In such contexts, women emphasize their heavy workload and note that confining gender norms such as …. disadvantage them in their career and can even plunge them and their households into poverty. In Andijan, middle class women reported that “the new development in the farming system which allows them to be become farm managers was not rapidly adopted by local people … It is always difficult for women to be as active as men because women usually have more duties than her job.” Poor women in Andijan likewise reported that “widespread traditional stereotypes like ‘a woman is a housekeeper’ and ‘the husband is a breadwinner’ hamper the development of women's professional careers.”

Equally important are norms related to women's participation in public life, on committees, and in public meetings which hinders their full functioning as independent farmers. This has changed in limited ways with women reaching subordinate roles in rural institutions such as accountant. Poor women in Bukhara, described as the most progressive challengers to traditional women's roles, explain that “according to local tradition it is forbidden to speak first among the management of *Mahalla* committee and at other meetings in *khokimiyat*. That is why a woman's farm is represented by a male relative. It is easier for him to talk with tractor drivers and men workers.” Members from this same group explain that such attitudes have negative implications for women's access to credit: “for women it is more difficult to apply to any state body or financial institutions because of negative attitudes of men to women.” Bukharan middle class women similarly explain that “women leaders and women farmers are hardly accepted because our men do not get used to sit with women in one place or, in other words, to take the same position as men. They are used to ordering women what to do and when to do it.”

Women in all four communities also expressed their dependence on Women's Committees in the *Mahalla* as important in order to access information about new wheat innovations. While Women's Committees in the *Mahalla* have proved to be a successful way for women to acquire information, it also reproduces gender norms related to women being subordinate to men. This is suggested by the fact that women who farm on a commercial basis were unable to become members in WUAs or acquire and lease agricultural machinery even as independent farmers. In fact, irrigation governance and access to machinery were areas of exchange that held most closely to traditional norms (see similar findings in Navbakhor Province reported by Gunchinmaa et al., 2011).

In all FGDs with middle class women, they explained that women innovators continue to need permission from their kin and husbands to take on farming roles beyond those that attend to the household plot. A young women's FGD in Bukhara noted that women have only recently begun to farm as independent producers and thus are considered to lack adequate knowledge of commercial cotton and wheat production. Women and men also reported that permission is often granted by the husband or male kin on condition that household duties are not compromised. This was indeed the case for a woman wheat farmer in Andijan who explained that her husband and in-laws were initially unwilling to support her farming but, eventually, given that she was able to meet her household responsibilities, they accepted her new farming role. Another Andijan woman reported ‘keeping quiet’ about her success in wheat cultivation because her husband was jealous. There is a similar story from a women farmer from Shodmonov:

“My husband will be jealous about extra attention and respect that are coming to his wife from other men. In our culture, men do not like when women are getting more respect, money, and attention. So, wife needs to be very accurate in communication with husband, such as do not show that she is the boss or the head of the family, it destroys family. I always appreciate my husband, and I never behave as if I am the great person, I just keep being a lovely wife. When you know the balance between wife and husband you may get the husband's trust and understanding. I have never forgotten that I am a wife and mother, so I prepare the food, wash the clothes and other home activities besides my job. He understands that I manage all house works, so he gives me freedom to work where I like” (UZ-Shodmonov-F2-F).

These findings are congruent with the literature on the need for women, in some situations, to subscribe to certain societal expectations in order to acquire certain freedoms (Cornwall and Edwards 2010) while highlighting women's multiple burdens or double and triple day (Cueva Beteta, 2006; Gartaula et al., 2010; Ibrahim and Alkire, 2007; Malhotra and Schuler, 2005).

Nonetheless, some of the women who farm on a commercial basis explained that they were able to enhance their household decision-making power as explained by an innovator woman interviewee in Bokhara: “now I decide with my husband, earlier he decided himself. Because he thought that I could be only housekeeper or a nurse with low salary. Only after gaining my own business and innovation practice [farming the land with improved wheat varieties] he believed that I could occupy an equal position and deserve respect as bread winner of family.” Most of the women farmers in our study areas indicated that women's role in decision making will only increase if she has been able to earn money from their business. Only then will their husbands support and listen to them. A woman farmer from Mugrak shared the following: “my husband retired on a pension because of health condition. So, I am the one to earn money for my family and I am doing good in my business. Now, he believes in my success, and started to help me to manage the farm, it was strong support” (UZ-Mugrak-G2-F). Other women explain that they too challenge gender norms. As one innovator farmer in Andijan reported,

“I proved that a man can listen to a woman. I see change in believing that women could succeed in getting the chance to grow seeds for other farmers meanwhile there were other men farmers who also would like to do this activity. I made man ??? authorities believe in our farm; I could get the trust of the workers and the Farmer-Sobir aka. They heard me, listened to me, consulted with me. I successfully negotiated with district authorities, and now men can address me at same level. My husband respects me more. I feel I gained this kind of approval that women also can manage the work that is done by men”.

Further, women whose husbands are migrants and who have undertaken new entrepreneurial activities, such as marketing or baking and selling special breads and foods, report that they have gained increased self-confidence: “My friends follow my example, baking from local seeds variety, they all need money … I am now more confident to try out new things” (a woman innovator in Andijan).

## 5 Conclusions

Our study indicates that in post-Soviet Uzbekistan, social and economic transitions have led to increased male outmigration resulting in the increased feminization of both farm labour and, to a lesser degree, farm management (World Bank report 2019). Women also are increasingly represented in wheat processing where they play a major role in refining, baking, and selling wheat products. While women feel that they have no choice but to be involved in leading these new activities, it has required them to negotiate changes in gender norms while simultaneous reproducing their traditional role in the household thereby increasing their work burden. Their expanded activities are supported by the government's interest in achieving self-sufficiency in wheat production and thus they do not discriminate between women and men in their access to required inputs, even as local communities are slower to provide women access to new resources and to recognize their equal participation in the governance of local agriculture and natural resource management. Our findings also reveal that the exclusive focus on wheat and cotton crops discriminates against women and stifles agricultural productivity by not providing them equal access to water and high-quality land. Our findings suggest that policy changes alone are insufficient to realize changes in gender expectations and highlights the need for innovators concerned with gender equality to work with local level households, extension officers, and village leaders to challenge gender norms that restrict women's full participation in all aspects of agricultural production. While evidence reveals that gender norms are still restrictive, it also shows that they are changing among mothers-in-law, especially in households with migrant males. Significantly, our data also reveals that in contexts that challenge norms women gain confidence and can more easily make demands for equal treatment. These changes are especially evident in migrant communities which reveal women to be forerunners of innovations in norms as well as in everyday social practice. Our findings also point to the *Mahallas* as changing environments for women's roles providing them with access to information and resources, even if in limited ways. Finally, gains incurred for women with male-outmigration and inclusion in *Mahallas* were at a cost of accentuating their double burden.

## Credit author statement

Dina Najjar: Conceptualization, Writing – original draft preparation. Rachana Devkota: Theoretical grounding, Writing, Editing. Shelley Feldman: Theoretical grounding, Writing, Editing.

## Funding

Funding obtained from CGIAR Research Program (CRP) Wheat (200181); CRP Policies, Institutions and Markets (200179); and the Bill and Melinda Gates Foundation (OPP1134630) have made this study possible.

## Footnotes

1 These migration rates are higher than reported in national and international statistics figures. Tursunova (2012) similarly raises concerns about the accuracy of migration statistics for an area in Khorezm where she was working. Migration numbers are difficult to determine especially due to illegal and seasonal migration (Asian Development Bank (ADB) 2014).

2 In general, women sell dairy products such as milk and yoghurt, as well as fruits and vegetables that are grown in their backyard and less often on rented land.
